# Feasibility of Novel Rear-Side Mirage Deflection Method for Thermal Conductivity Measurements

**DOI:** 10.3390/s21175971

**Published:** 2021-09-06

**Authors:** Gwantaek Kim, Moojoong Kim, Hyunjung Kim

**Affiliations:** 1 Department of Mechanical Engineering, Ajou University, Suwon 16499, Korea; scomi02@ajou.ac.kr; 2 SAITO LAB, Waseda Research Institute for Science and Engineering, Waseda University, Shinjuku-ku, Tokyo 169-8555, Japan; mj_kim@aoni.waseda.jp

**Keywords:** photothermal effect, phase delay, rear-side mirage method, light absorption thin film, thermal conductivity, thermal properties

## Abstract

Among the noncontact measurement technologies used to acquire thermal property information, those that use the photothermal effect are attracting attention. However, it is difficult to perform measurements for new materials with different optical and thermal properties, owing to limitations of existing thermal conductivity measurement methods using the photothermal effect. To address this problem, this study aimed to develop a rear-side mirage deflection method capable of measuring thermal conductivity regardless of the material characteristics based on the photothermal effect. A thin copper film (of 20 µm thickness) was formed on the surfaces of the target materials so that measurements could not be affected by the characteristics of the target materials. In addition, phase delay signals were acquired from the rear sides of the target materials to exclude the influence of the pump beam, which is a problem in existing thermal conductivity measurement methods that use the photothermal effect. To verify the feasibility of the proposed measurement technique, thermal conductivity was measured for copper, aluminum, and stainless steel samples with a 250 µm thickness. The results were compared with literature values and showed good agreement with relative errors equal to or less than 0.2%.

## 1. Introduction

With the development of different industries, many new materials have been developed and are being applied to various products, such as semiconductors, optical devices, MEMS devices, and displays. In particular, the miniaturization of the processes applied to fabricate semiconductors in various industries has led to high integration and performance. In semiconductor fabrication processes, layers composed of electronic circuits in various forms are present on a wafer, which is a single-crystal thin film of silicon (Si) or gallium arsenide (GaAs) [1]. Each layer is composed of representative materials with various optical and thermal properties, as mentioned in Table 1 [2,3,4]. The optimal thermal design of semiconductors is becoming difficult, owing to the miniaturization of the processes and diversification of the materials constituting electronic circuits [5]. For the optimal thermal design of semiconductors, the thermal property information of materials with various properties is required.

Thermal property measurements are generally performed by analyzing the temperature variation pattern of a material as a function of the heat input. Methods for thermal property measurement can be divided into contact and noncontact types depending on the heat input and temperature measurement methods. Table 2 presents the application ranges and limitations of these types of methods [6,7,8,9,10,11,12,13,14,15]. For the contact type, heat input and temperature measurements are performed while the material is in contact with the measuring device. In this process, a relatively large error occurs owing to the contact resistance. Moreover, it is difficult to attach sensors to devices that require airproof, and materials can be damaged during measurements. However, the noncontact type has relative benefits in terms of the contact resistance caused by contact with materials, sensor attachment, and material damage because the heat input or temperature measurements are performed in a noncontact manner. Based on these benefits, studies have been conducted on various noncontact methods. Among them, the thermal property measurement method using the photothermal effect is the most advanced method, and its measurement reliability has been validated in several studies [16,17,18,19,20,21,22]. Recent thermophysical properties measurement techniques have been applied to nanofluids beyond bulk solids and thin films [23]. In particular, nanofluid particle properties, concentration, and size significantly affect the sample’s photothermal effect and thermal profile formation. Therefore, thermal properties measurement technology using the photothermal effect, which has advantages in microspectroscopic technology, is in the spotlight [24,25,26,27].

**Figure 1:**
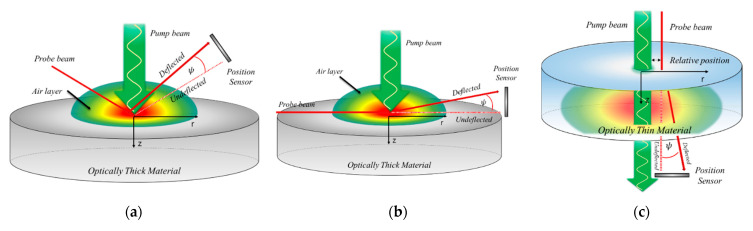
Thermal conductivity measurements using photothermal effect. (**a**) Displacement method, (**b**) mirage deflection method, and (**c**) collinear deflection method.

The equipment for thermal property measurements using the photothermal effect consists of a pump beam for providing the heat input and a probe beam for temperature change measurements. Depending on the properties of the materials to be measured and alignment of the pump and probe beams, the measurement approaches are classified into the displacement method, mirage deflection method, and collinear method, as shown in Figure 1.

The displacement measuring method was established by Olmstead et al. [28]. Ospal [29,30] and Jeon [31] conducted one-dimensional and three-dimensional theoretical analyses of the surface temperature and thermoelastic deformation. Lee [32] and Jeon [33] experimentally measured the thermal diffusivity. For the mirage deflection method, Salazar et al. [16] established a thermal property measurement method for monolayer isotropic materials, and Jeon et al. [34,35] studied the measurement of the thermal properties of monolayer anisotropic materials. For the collinear deflection method, Salazar [36,37], Spear [17,38], and Kim [39] conducted research on materials with transparent single/double layer structures.

For thermal property measurement methods using the photothermal effect, the thermoelastic deformation of the specimen heated by the periodic pump beam, refractive index change (due to the density change) in the air layer adjacent to the specimen, and refractive index change (due to the density change) inside the specimen are used. The displacement method utilizes the temperature increase-induced thermoelastic deformation of the specimen irradiated by the pump beam, as shown in Figure 1a. As the thermal properties are measured using the change in the reflection angle of the probe beam caused by thermoelastic deformation, the uniformity of the specimen surface reflectivity has a large impact. In other words, the accuracy of thermal property measurements differ depending on the uniformity of the specimen surface. Moreover, measurement is difficult for transparent materials with low light absorption. The mirage deflection method utilizes the change in the refractive index of the air layer adjacent to the specimen. As shown in Figure 1b, the pump and probe beams are aligned perpendicular to each other to measure the thermal properties of the target material. This method exhibits high accuracy when the target material is opaque (low transmittance); the energy of the pump beam is sufficiently absorbed and a sufficient temperature gradient can be generated in the air layer adjacent to the specimen. Therefore, it is difficult to measure the thermal properties of target materials with high transmittance using the mirage deflection method. To overcome this drawback, the thermal properties of a material with high transmittance were measured using the collinear deflection method, as shown in Figure 1c. The collinear deflection method uses changes in the refractive index of the air layer and material by aligning the probe beam parallel to the pump beam to measure the thermal properties of a material with low pump beam energy absorption (i.e., high transmittance). However, this method produces a large measurement error because materials with a transmittance higher than a certain level do not undergo refractive index changes sufficient for measurement. Existing thermal property measurement methods using the photothermal effect have a common problem, in that a phase delay error occurs owing to mutual interference in the area where the pump and probe beams cross [40]. In summary, existing thermal property measurement methods using the photothermal effect depend on the thermal and optical properties of materials. Therefore, measurements are not possible for materials that do not possess optical and thermal properties satisfying the measurement conditions of each method.

**Figure 2:**
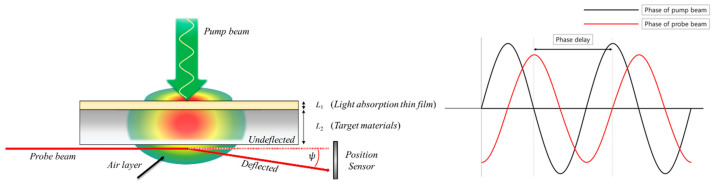
Schematic of the rear-side mirage deflection method and an example showing phase difference derivation.

To overcome the limitations of the existing thermal property measurement methods using the photothermal effect, this study attempted to analyze the rear-side mirage deflection method, a new thermal property measurement method that also uses the photothermal effect. The proposed rear-side mirage deflection method has a light absorption thin film made of copper, as shown in Figure 2; the probe beam is incident in the rear side of the target material to measure the phase delay. A detailed explanation is provided in Section 2. The copper film can sufficiently absorb the energy of the pump beam with a low thickness of 20 μm because it has a high optical absorption coefficient. This is a very important factor in the thermal property measurement technology proposed in this study; as the energy of the pump beam is sufficiently absorbed by a light absorption thin film, this thermal property measurement method can be applied regardless of the optical properties of the target materials. Moreover, it is expected that placing the probe beam on the rear side of the specimen will improve the accuracy of the thermal property measurement results by preventing the crossing of the pump and probe beams, which causes a large phase delay error in the existing thermal property measurement methods using the photothermal effect.

The rear-side mirage deflection method is a novel thermal property measurement method that has not been proposed before. Thus, it is necessary to carefully examine the applicability of this method before utilizing it for analyzing materials with various optical and thermal properties. To this end, the temperature distributions of specimens with a double-layer structure, including a light absorption thin film and each of the target materials, were derived through theoretical analysis using a three-dimensional unsteady heat conduction equation. Based on this equation, deflection analysis of the probe beam passing through the rear side of each specimen was obtained, and the phase delay was calculated according to the relative position. The relative position is the relative distance between the pump beam and the probe beam in the r-direction (or x-direction) direction as shown in Figure 1c. To verify the results, 270 µm-thick specimens with a 20-μm-thick light absorption thin film made of copper were fabricated by electrodeposition. Copper, aluminum, and stainless steel (SUS 304) that have well-known thermal conductivities were used to fabricate the specimens. For the three specimens, phase delays were measured according to the relative positions and compared with the phase delay derived through theoretical analysis to determine the thermal conductivity. Based on this measurement, the feasibility of the proposed rear-side mirage deflection method was assessed.

## 2. Background Theory

### 2.1. Rear-Side Mirage Deflection Method

The photothermal effect used in the proposed method is the phenomenon in which light energy is converted into thermal energy through the increased energy level on the material surface when the photons of the pump beam collide with the material surface. The setup for implementing the rear-side mirage deflection method is constructed as shown in Figure 2 to overcome the limitations of existing thermal property measurement methods. The specimen consists of the two layers (i.e., the light absorption thin film and target material); the probe beam passes through the air layer beneath the specimen to avoid mutual interference between the pump and probe beams. The area of the light absorption thin film is sufficient to absorb the energy of the pump beam regardless of the optical and thermal properties of the target material. The optical absorption coefficient (λ) of copper is $6.1310\times 10^{5}{\;\mathrm{cm}}^{-1}$ [41], and the entire energy of the pump beam is absorbed on the surface of the light absorption thin film because the optical absorption length ($L_{abs}$) is very short (16.31 nm) according to Equation (1).

Therefore, all of the absorbed energy is converted to heat in the light absorption thin film; this causes a periodic temperature gradient in the target material and the upper/lower air layers through conduction. Owing to this physical phenomenon, the thermal properties of target materials can be measured using the photothermal effect regardless of their optical properties. When the probe beam is passed through the air layer beneath the specimen with a periodic temperature gradient, deflection occurs owing to the temperature gradient. In this case, the deflection angle with respect to the traveling path of the probe beam (Φ) can be expressed as:

(1) $$L_{abs}=\frac{1}{\lambda }$$

(2) $$\Phi =\int_{path}^{}\frac{1}{n}\left(\frac{\partial n}{\partial T}\right)\nabla_{n}T\left(x,y,z,t\right)ds$$

where n is the refractive index, ∂n/∂T is the temperature coefficient of the refractive index, and $\nabla_{n}T$ is the temperature gradient perpendicular to the probe beam path.

As the pump beam is modulated periodically, the temperature gradient beneath the specimen repeatedly increases (heating) and decreases (cooling) according to the period of the pump beam. In this case, a time delay occurs between the temperature gradient and period of the pump beam depending on the thermal properties of the light absorption thin film and the target material. As a result, there is a time delay between the period of the pump beam and that of the deflection of the probe beam passing through the temperature gradient shown in Figure 2, which is referred to as the phase delay. This phase delay increases as the relative position between the pump and probe beams increases; it varies depending on the thermal properties of the material. Based on this principle, the thermal conductivity of the target material can be derived if the phase delay curve is analyzed according to the relative position.

### 2.2. Temperature Distribution and Phase Delay Analysis

**Figure 3:**
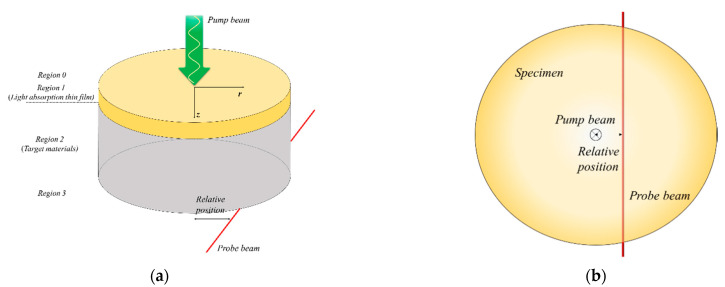
Schematic of the rear-side mirage deflection method. (**a**) Diametric view and (**b**) top view.

The phase delay between the pump beam and probe beam was experimentally measured through the rear-side mirage deflection method. To calculate the phase delay through theoretical analysis, information on the temperature distribution is required. To obtain this information, the 3D unsteady state heat conduction equation with a periodic heat source (Equation (3)) was analyzed in this study. The influence of convection and radiation was neglected because the temperature increase in the specimen due to the pump beam was small. Figure 3 shows the target material model with a light absorption thin film. The axis perpendicular to the specimen surface was set as the *z*-axis. Regions 0 and 3 are air layers around the specimen where light absorption is not significant. Region 1 represents the light absorption thin film, and region 2 is the target material.

(3) $$\nabla^{2}T_{i}-\frac{1}{\alpha_{i}}\frac{\partial T_{i}}{\partial t}=-\frac{Q_{i}}{k_{i}}\;(i=\mathrm{front}\;\mathrm{air}\;(0),\;\mathrm{light}\;\mathrm{absorption}\;\mathrm{thin}\;\mathrm{film}\;(1),\;\mathrm{target}\;\mathrm{material}\;(2),\;\mathrm{rear}\;\mathrm{air}\;(3))$$

(4) $$Q_{1}\left(r,z,t\right)=\frac{\lambda_{1}P_{1}}{4\pi a^{2}}e^{-\frac{r^{2}}{a^{2}}-\lambda_{1}z}\left[1+\cos\left(\omega t\right)\right]$$

(5) $$Q_{2}\left(r,z,t\right)=\frac{\lambda_{2}P_{2}}{4\pi a^{2}}e^{-\frac{r^{2}}{a^{2}}-\lambda_{2}\left(z-L_{1}\right)-\lambda_{1}L_{1}}\left[1+\cos\left(\omega t\right)\right]=0$$

(6) $$P_{1}=P_{0}\left(1-R_{1}\right)$$

(7) $$P_{2}=P_{0}\left(1-R_{1}\right)\left(1-R_{2}\right)$$

where $Q_{1}\left(r,z,t\right)$ and $Q_{2}\left(r,z,t\right)$ represent the heat sources in regions 1 and 2; $\lambda_{1}$ and $\lambda_{2}$ represent the optical absorption coefficients of each region; and $R_{1}$ and $R_{2}$ denote the reflectance at the interface between regions 0 and 1 and between regions 1 and 2, respectively. As the light absorption thin film absorbs all the heat provided by the heat source, the heat source in region 2, which is the region of the target material, can be considered zero. If Equations (4)–(7) are substituted into Equation (3), Equations (8)–(11) are obtained:

(8) $$\nabla^{2}T_{0}-\frac{1}{\alpha_{0}}\frac{\partial T_{0}}{\partial t}=-\frac{Q_{0}}{k_{0}}=0$$

(9) $$\nabla^{2}T_{1}-\frac{1}{\alpha_{1}}\frac{\partial T_{1}}{\partial t}=-\frac{Q_{1}}{k_{1}}=-\frac{\lambda_{1}P_{1}}{4\pi k_{1}a^{2}}e^{-\frac{r^{2}}{a^{2}}-\lambda_{1}z}\left[1+\cos\left(\omega t\right)\right]$$

(10) $$\nabla^{2}T_{2}-\frac{1}{\alpha_{2}}\frac{\partial T_{2}}{\partial t}=-\frac{Q_{2}}{k_{2}}=0$$

(11) $$\nabla^{2}T_{3}-\frac{1}{\alpha_{3}}\frac{\partial T_{3}}{\partial t}=-\frac{Q_{3}}{k_{3}}=0.\;$$

As observed in Equations (12)–(14), boundary conditions in which the temperature and heat flux at the interface between each region are the same were applied:

(12) $$\begin{array}{cc} {T_{0}|}_{z=0}={T_{1}|}_{z=0} & k_{0}{\frac{\partial T_{0}}{\partial z}|}_{z=0}=k_{1}{\frac{\partial T_{1}}{\partial z}|}_{z=0} \end{array}$$

(13) $$\begin{array}{cc} {T_{1}|}_{z=L_{1}}={T_{2}|}_{z=L_{1}} & k_{1}{\frac{\partial T_{1}}{\partial z}|}_{z=L_{1}}=k_{2}{\frac{\partial T_{2}}{\partial z}|}_{z=L_{1}} \end{array}$$

(14) $$\begin{array}{cc} {T_{2}|}_{z=L_{1}+L_{2}}={T_{3}|}_{z=L_{1}+L_{2}} & k_{2}{\frac{\partial T_{2}}{\partial z}|}_{z=L_{1}+L_{2}}=k_{3}{\frac{\partial T_{3}}{\partial z}|}_{z=L_{1}+L_{2}} \end{array}$$

The complex transformation method was applied to convert the unsteady state heat conduction equation to a steady state equation and easily derive the deflection angle of the probe beam. When the boundary conditions were substituted after Hankel transformation, the temperature equation for each region could be obtained as shown in Equations (15)–(18) [35]. A detailed description of Hankel transformation can be found in Appendix A.

(15) $$T_{0}=\int_{0}^{\infty }\left\{C_{1}e^{\delta_{0}z}\right\}\beta J_{0}\left(\beta r\right)e^{i\omega t}d\beta$$

(16) $$T_{1}=\int_{0}^{\infty }\left\{A_{1}e^{-\lambda_{1}z}+C_{3}\cosh\left(\delta_{1}z\right)+C_{4}\sinh\left(\delta_{1}z\right)\right\}\beta J_{0}\left(\beta r\right)e^{i\omega t}d\beta$$

(17) $$T_{2}=\int_{0}^{\infty }\left\{A_{2}e^{-\lambda_{2}\left(z-L_{1}\right)}+C_{5}\cosh\left(\delta_{2}\left(z-L_{1}\right)\right)+C_{6}\sinh\left(\delta_{2}\left(z-L_{1}\right)\right)\right\}\beta J_{0}\left(\beta r\right)e^{i\omega t}d\beta$$

(18) $$T_{3}=\int_{0}^{\infty }\left\{C_{8}e^{-\delta_{3}\left(z-L_{1}-L_{2}\right)}\right\}\beta J_{0}\left(\beta r\right)e^{i\omega t}d\beta$$

Here, $J_{0}$ is the Bessel function of the first kind, and the coefficients are as follows:

$$A_{1}=-\frac{\lambda_{1}P_{1}e^{-\frac{\beta^{2}a^{2}}{4}}}{8\pi k_{1}\left(\lambda_{1}^{2}-\delta_{1}^{2}\right)}$$

$$A_{2}=-\frac{\lambda_{2}P_{2}e^{-\lambda_{1}L_{1}-\frac{\beta^{2}a^{2}}{4}}}{8\pi k_{2}\left(\lambda_{2}^{2}-\delta_{2}^{2}\right)}$$

$$C_{1}=A_{1}+C_{3}$$

$$C_{4}=\left[\chi_{0}C_{1}+k_{1}\lambda_{1}A_{1}\right]/\chi_{1}$$

$$C_{5}=-A_{2}+A_{1}e^{-\lambda_{1}L_{1}}+C_{3}\cosh\left(\delta_{1}L_{1}\right)+C_{4}\sinh\left(\delta_{1}L_{1}\right)$$

$$C_{6}=k_{2}\lambda_{2}A_{2}-A_{1}k_{1}L_{1}e^{-\lambda_{1}L_{1}}+C_{3}\chi_{1}\sinh\left(\delta_{1}L_{1}\right)+C_{4}\chi_{1}\cosh\left(\delta_{1}L_{1}\right)/\chi_{2}$$

$$C_{8}=\left[-A_{2}k_{2}\chi_{2}e^{-\lambda_{2}L_{2}}+C_{5}\chi_{2}\sinh\left(\delta_{2}L_{2}\right)+C_{6}\chi_{1}\cosh\left(\delta_{2}L_{2}\right)\right]/\chi_{3}$$

$$H_{1,2}=\left[e^{-\delta_{2}L_{2}}\left(\chi_{2}+\chi_{3}\right)\pm e^{\delta_{2}L_{2}}\left(\chi_{2}-\chi_{3}\right)\right]/2$$

$$\Gamma_{1,2}=\left[e^{-\delta_{1}L_{1}}\left(\chi_{1}H_{1}+\chi_{2}H_{2}\right)\pm e^{\delta_{1}L_{1}}\left(\chi_{1}H_{1}-\chi_{2}H_{2}\right)\right]/2$$

$$\delta_{i}^{2}=\beta^{2}+i\omega /\alpha_{i}$$

$$\chi_{i}=\delta_{i}k_{i}\;\;\;\;\;\;\;\;\;\;\;\left(i=0,1,2,3\right)$$

The deflection angle of the probe beam was calculated using the temperature gradient for the probe beam path, as in Equations (2) and (3). As shown in Equation (19), the deflection angle is calculated along the direction normal to the probe beam path using the temperature gradient of the air layer beneath the specimen (region 3).

(19) $$\Phi =\frac{1}{n_{3}}\left(\frac{\partial n_{3}}{\partial T_{3}}\right)\int_{-\infty }^{\infty }\frac{\partial T_{3}}{\partial z}dr$$

As the deflection analysis results are composed of complex numbers, the phase delay (ψ) between the probe beam and pump beam can be expressed as:

(20) ψ=tan−1Im(Φ)Re(Φ)

### 2.3. Thermal Property Determination Algorithm

**Figure 4:**
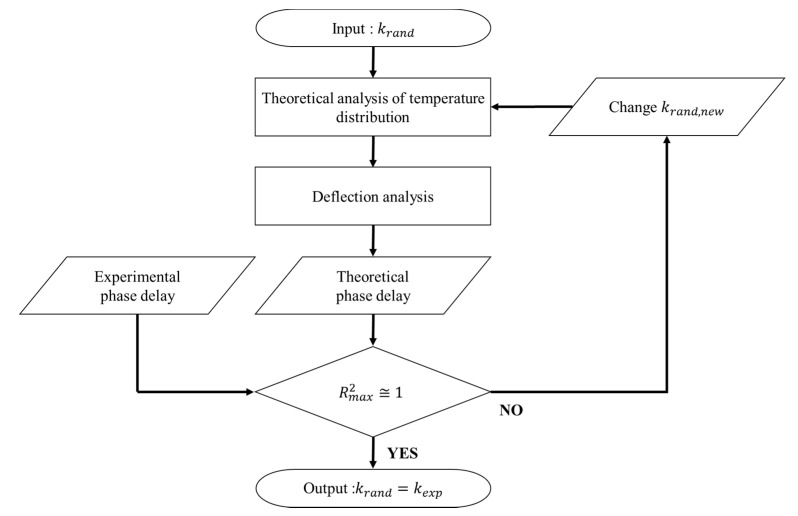
Flowchart of the thermal conductivity algorithm used in the rear-side deflection method.

As explained above, the phase delay was determined experimentally and theoretically according to the relative position between the pump and probe beams. However, as the actual thermal conductivity of the target material is not known, an algorithm to determine the thermal conductivity is required. The representative methods include the phase gradient method, zero-crossing method, and phase curve method. In this study, the algorithm to determine the thermal conductivity based on the phase curve method was used, as shown in Figure 4. The phase curve method is used to determine the actual thermal conductivity by comparing the theoretical phase delay ($\psi_{theo}$) according to the relative position derived from the random thermal conductivity ($k_{rand}$) and experimental phase delay ($\psi_{exp}$) according to the relative position using the coefficient of determination. It determines the similarity using the least squares method, a statistical method, using Equation (21). The least squares method finds a solution that minimizes the sum of the squares of the errors (SSE) between the approximate solution and actual solution. As shown in Equation (21), the SSE means the sum of squares of the difference between the measured value $Y_{i}$ and estimated value ${\hat{Y}}_{i}$ at each relative position point. The sum of the squares due to regression (SSR) represents the sum of squares of the difference between the estimated value ${\hat{Y}}_{i}$ and the average value $\bar{Y}$. The total sum of the squares (SST) is expressed as the sum of SSE and SSR. Theoretically, it can be determined that the error is smaller as the coefficient of determination is closer to 1. In this study, ${\hat{Y}}_{i}$ is the phase delay ($\psi_{theo}$) at each relative position point derived through theoretical analysis; $\bar{Y}$ represents the average value of the phase delay measured experimentally (${\bar{\psi }}_{exp}$) and $Y_{i}$ is the phase delay ($\psi_{exp}$.) at each relative position point, also measured experimentally.

(21) $$R^{2}=1-\frac{SSE}{SST}\;SSE=\sum^{\;}{\left({\hat{Y}}_{i}-Y_{i}\right)}^{2}=\sum^{\;}{\left(\psi_{theo}-\psi_{exp}\right)}^{2}\;SSR=\sum^{\;}{\left({\hat{Y}}_{i}-\bar{Y}\right)}^{2}=\sum^{\;}{\left(\psi_{theo}-{\bar{\psi }}_{exp}\right)}^{2}\;SST=SSE+SSR$$

## 3. Experimental Investigation

### 3.1. Specimens Used for Experiments

The purpose of this study was to develop a universally applicable thermal property measurement technique using the photothermal effect and to verify its applicability. To this end, specimens in which a light absorption thin film (a metal thin film with high light absorption) was added to the surfaces of the target material were used. The 20 µm-thick light absorption thin film made of copper was formed through electrodeposition. Electrodeposition was conducted on three specimens (copper, aluminum, and SUS) to produce a thin film for light absorption. To this end, electroplating was conducted using a copper sulfate (CuSO_4_) solution to supply current to the generator and regulate the current supplied through the bias resistor. When a cathode is connected to the object to be measured and power is supplied, electrons emitted from the cathode are transferred to the copper ions in the solution to precipitate copper. The precipitated copper accumulates at the normal atomic spacing on the surface of the measurement target substance connected to the negative electrode. In addition, the number of sulfate ions generated is similar to the number of copper atoms precipitated, and they stick to the surface of the anode; hence, the efficiency of the cathode and the anode becomes equal to 100% without any side reaction or loss. As the thermal properties of the light absorption thin film were required to derive the phase delay through theoretical analysis, the composition of the film was analyzed through X-ray fluorescence (XRF) spectroscopy using a ZSX Primus spectrometer from Rigaku; XRF analysis using an X-ray tube of end-window type Rh target with voltage in the range 40–60 kV was conducted, and the sample was irradiated to excite the trajectory electrons of its constituent elements. Electrons in the excited state return to the ground state, and during continuous electron rearrangement, fluorescent X-rays are emitted according to the characteristics of the elements constituting the sample; the intensity of the X-ray diffracted at a certain angle can be quantitatively measured for each element using a detector. The XRF results confirmed that 99.95% of the film was copper (Table 3). For the thermal properties of the light absorption thin film, the thermal properties of copper were used (Table 4).

The purpose of this study was to verify the applicability of the proposed method, and thus it was necessary to select commonly used materials whose accurate thermal properties are known as target materials (Table 4). Therefore, three opaque and metallic materials (copper, aluminum, and SUS) with different thermal properties were selected; their thermal properties are listed in Table 4. The target materials were in wafer (copper, aluminum, and SUS sheets) form with a thickness of 250 µm and diameter of 50.8 mm. Copper and aluminum were procured from Sigma Aldrich and SUS 304 was purchased from Nilaco Corporation. Three samples were used for each material, and the average value was used by performing the phase delay measurement three times per sample.

### 3.2. Experimental Setup for Rear-Side Mirage Deflection Method

**Figure 5:**
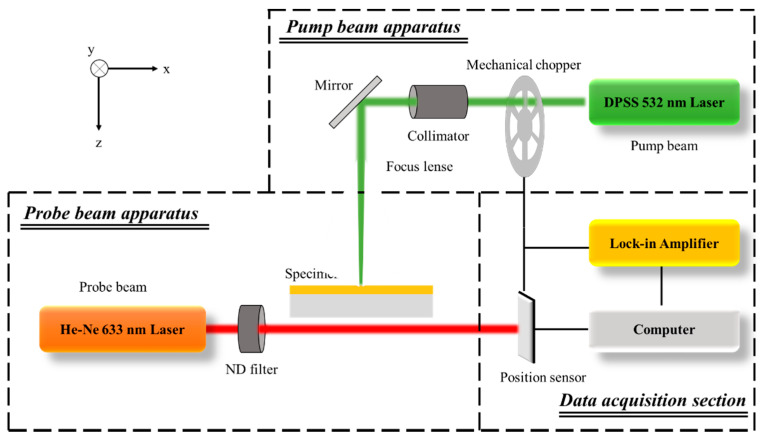
Schematic of optical alignment in the rear-side mirage deflection method.

Figure 5 shows a schematic of the experimental setup and optical alignment. The place where the experimental equipment was set up was a constant temperature chamber with a size of 6 × 6 m, and an optic table was set up in the center. The optic table used the DVIO-R-3012M-200t (800H) model from DAEIL SYSTEMS, and the experimental equipment was configured as shown in Figure 5 on the optic table. When clamping the specimen, consideration must be given to whether the path of the probe beam is obstructed, and the 2 inch wafer should be fixed so that it does not quiver. The specimen was fixed using the Thorlabs FH2 model, which satisfies the corresponding conditions. For phase delay measurement using photothermal effect, ambient temperature and humidity are important. Therefore, an air conditioner was used to maintain an internal temperature of 25 °C and a humidity of 30–40%. For the pump beam, A Sprout-G continuous-wave, diode-pumped solid-state (DPSS) source containing a 532 nm laser with a Gaussian distribution was used. To give periodic heating, a sinusoidally modulated frequency was produced using a mechanical chopper from Stanford Research Systems (SRS). Periodic heating of 20 Hz was applied to the front side of the specimen. A pump beam was passed through the collimator of Newport’s LC-075 model and made parallel, and then passed through a focus lens with a focal length of 500 mm. For the probe beam, a Newport He–Ne laser with a wavelength of 633 nm and power of 5 mW was used. Newport’s ULM-TILT model was used for aligning the probe beam, and it was connected to the same stage as the photoelectric position sensor. The relative position between the pump beam and probe beam was adjusted to 100 μm using a Newport M-TS50DC.5 motorized stage. The deflection angle of the probe beam occurring because of the temperature gradient in the air layer was measured using a C100443-01 photoelectric position sensor from Hamamatsu Photonics. Through the comparison of the deflection angle of the probe beam measured using the position sensor and the modulation frequency of the mechanical chopper, the phase delay was derived using an AMETEK 7270 DSP lock-in amplifier.

Figure 3 and Figure 5 show the detailed alignment of the specimen, pump beam, and probe beam. The intensity and radius of the pump beam, height of the probe beam, relative position between the pump beam and the probe beam, and modulation frequency of the pump beam used are shown in Table 5. For the relative position between the pump and probe beams, the 0–0.15 mm section cannot be used in existing thermal property measurement methods using the photothermal effect, owing to the interference between the pump and probe beams [39]. However, in the proposed method, it is possible to obtain the phase delay using the entire section from 0 to 0.6 mm because there is no interference due to the specificity of the optical alignment. For the phase delay, approximately 300 results were obtained from a relative position for 30 s after the temperature difference reached a quasi-steady state through periodic heating with a mechanical chopper. Among them, the data in the 95% confidence interval were averaged arithmetically and the results were used as representative values, and outliers were confirmed to be less than 10% at all measurement points. The noise that may occur in each measurement was filtered through a lock-in amplifier to secure measurement reliability. In addition, since the pump beam and the probe beam are physically isolated by the specimen, noise generation factors such as laser mutual interference are reduced, unlike in other existing methods using the photothermal effect.

## 4. Results

**Figure 6:**
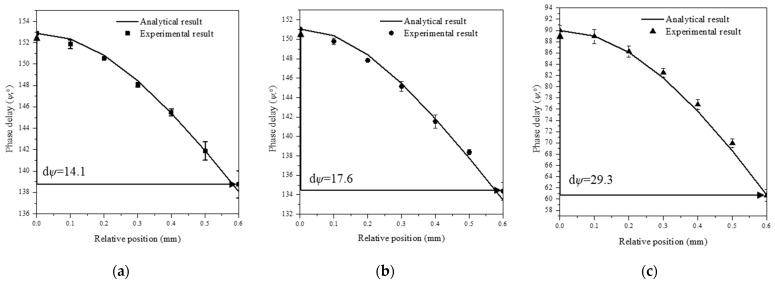
Phase delays derived from experimental and theoretical analysis. (**a**) Copper, (**b**) aluminum, and (**c**) stainless steel.

Before determining the thermal conductivity using the thermal conductivity determination algorithm, it was necessary to check whether the experiment and theoretical analysis were conducted properly. Thus, the phase delay results were derived according to the relative position obtained through theoretical analysis using the properties of the target materials (Table 4). The results are shown together with the experimental results in Figure 6. The error bar of the experimental data at each measurement point (relative position point) is the standard deviation of the mean value of the data with a 95% confidence interval for approximately 300 results obtained at the relative position point for three samples. For the three target materials, it was found that the phase delay results derived through theoretical analysis agreed well with those derived experimentally. For copper with a high thermal conductivity, the phase delay changed gradually with the relative position, and for stainless steel with a low thermal conductivity, the phase delay changed abruptly with the relative position. The contact resistance between the light absorption thin film and the target materials that occurs during electrodeposition may cause errors when measuring the thermal conductivities of the target materials. The influence of contact resistance was indirectly determined from the results (Figure 6a) obtained via theoretical analysis and experiments for the case where both the light absorption thin film and target material were copper (light absorption thin film thickness: 20 µm; target material thickness: 250 µm; total thickness: 270 µm). As described in the temperature distribution and phase delay analysis, the target used in theoretical analysis was composed of double layers, but the contact resistance that could occur between the layers was not considered.

Nevertheless, the results in Figure 6a show that the analytical results are in good agreement with the experimental results with relative errors less of than 0.5%. This indicates that the influence of contact resistance during the electrodeposition process is very small. At all measurement points in Figure 6, it was confirmed that the error bars were measured within 3.7%, the result of the uncertainty analysis included in Appendix B.

**Figure 7:**
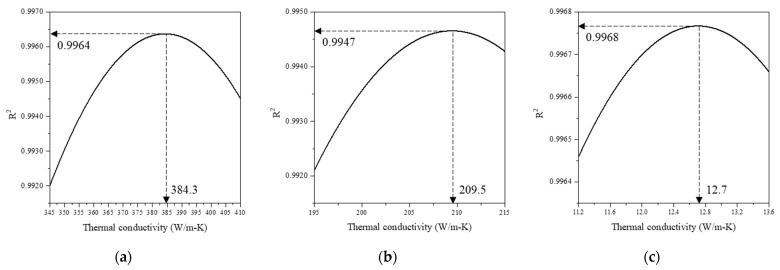
Thermal conductivity maximizing the coefficient of determination. (**a**) Copper, (**b**) aluminum, and (**c**) stainless steel.

Changes in the coefficient of determination according to the randomly substituted thermal conductivity values shown in Figure 7 were obtained by applying the phase curve algorithm. As observed in Figure 7, there exists a point that maximizes the coefficient of determination. In this case, the substituted thermal conductivity value was determined as the thermal conductivity of the target material. The results exhibited relative errors equal to or less than 0.2% when compared with the actual thermal conductivities of the target materials (Table 4). Table 6 presents the results along with the actual thermal conductivities. For copper, the error was significantly reduced, compared to the previous thermal property measurement error; 2.1% in mirage deflection method [34]. As mentioned in the introduction, the reason is that the mirage deflection method has been used in the previous study that reported that the laser interference occurred at the relative position 0 mm, where the pump beam and the probe beam overlap.

## 5. Conclusions

In this study, a rear-side mirage deflection method that uses a thin copper film capable of absorbing all the energy of the pump beam was developed; to solve the problem of interference between the pump beam and the probe beam, which is a common problem of existing measurement methods using photothermal effect, the pump beam was irradiated to the front side and the probe beam was irradiated to the rear side of the specimen. To verify the proposed method, thermal property measurements were performed for three opaque and metallic target materials whose thermal properties are well known. The three target materials were selected as copper, aluminum, and stainless steel with various thermal conductivity within 0.2% compared to reference values. Through this, not only did the measurement accuracy improve, but also the possibility of measuring the thermal conductivity coefficient regardless of the thermal and optical properties of the target material was confirmed. Due to the characteristics of the method used to measure from the rear side of the specimen, the specimen should be made as thin as mm or less, but this is not a thickness that is difficult to manufacture. The following are the conclusions of the study.

(1) For the rear-side mirage deflection method, a three-dimensional unsteady state heat conduction equation was established, and the three-dimensional unsteady state temperature distribution was derived using the complex transformation method and Hankel transformation. The theoretical phase delay between the pump beam and the probe beam was derived via temperature distribution to conduct the deflection analysis of the probe beam.
(2) A light absorption thin film made of copper was formed on each of the target materials using the electrodeposition process to eliminate the influence of the optical properties of the target materials. The purity of the light absorption thin film was measured using XRF, and it was found that approximately 99.95% of the film was copper. The influence of the contact resistance between the light absorption thin film and the target materials could be indirectly examined in the case where the target material was copper. The theoretical phase delay results, which did not consider the contact resistance, showed good agreement with the experimental phase delay results with relative errors of less than 0.5%. This indicates that the influence of the contact resistance between the light absorption thin film and the target materials on the phase delay is not significant.
(3) Thermal conductivity could be determined by applying the phase curve algorithm to the experimentally measured phase delay results. The determined thermal conductivities of the three target materials (copper, aluminum, and stainless steel) showed good agreement with those provided by the manufacturers with relative errors equal to or less than 0.2%. This indicates that the accuracy was significantly improved considering that the relative error obtained in the existing study, which performed measurements through the mirage deflection method, was 2.1%.

In this study, the wide applicability of the rear-side mirage deflection method was confirmed through conductivity measurements of copper, aluminum, and stainless steel, whose properties are well known. In addition, as there is no interference between the pump and probe beam, it is considered that it can be applied to materials that are difficult to surface treatment and materials with very high light transmittance. However, a parametric study on the thickness of the light absorption thin film was not conducted, and the measurement was performed only on opaque and metallic materials; this is a limitation. Future work is needed to examine various other parameters (e.g., thickness, material, and formation method of the thin film).

## Figures and Tables

**Table 1:** Materials constituting semiconductors and their properties.

Category	Materials	Thermal Conductivity (W/m-K)	Refractive Index
Substrate	B, C_diamond_, Si, P, Ga, GaAs, Se, Sn, and Te	0.05–2.90 × 10^−3^	2.3–3.97
Metal	Ag, Au, Cu, Al, Carbon Nanotubes, and Graphene	210–419	0.5–0.9

**Table 2:** Limitations of contact-type and noncontact-type thermal property measurement technologies.

	Method	Limitation
Contact	DC heating [6,7,8]	Conductive materials only
	Pulse heating [9,10,11]	Conductive materials only
	Laser calorimetry [12]	Large amount of heat loss
	3ω Method [13]	Complicated method causing specimen damage
Noncontact	Photo Acoustic [12]	Low accuracy
	Laser flash [14,15]	Specimen damage and limitations in terms of sample shape/size
	Photothermal radiometry [12]	Problem of emissivity factor
	Photothermal reflection [12]	Standardization of roughness on surface of specimen
	Photothermal displacement [12]	Surface treatment of specimen
	Photothermal deflection [12]	Increase in S/N ratio

**Table 3:** Composition of the light absorption thin film.

Element	Proportion (m%)
Cu	99.9461
S	0.0223
P	0.0316

**Table 4:** Properties of experimental target materials.

Material	Thermal Conductivity (W/m-K)	Specific Heat (J/kg K)	Density (kg/m^3^)
Copper [42,43]	385	385	8960
Aluminum [3,44]	210	900	2699
SUS 304	12.7	500	7945

**Table 5:** Experimental optical conditions.

	Condition	Errors	
Intensity of pump beam (W)	10	Power stability	±0.25% rms
Radius of pump beam (μm)	75	2.3 mm ± 10%	
Height of probe beam (μm)	150	±3 μm	
Relative position (mm)	0 to 0.6 (interval 0.1)	±1.2 μm	
Modulated frequency (Hz)	20	<2%	

**Table 6:** Experimental results.

Material	$k_{ref}$ (W/m-k)	$k_{exp}$ (W/m-k)	Relative Error (%)
Copper	385	384.3	0.1
Aluminum	210	209.5	0.2
SUS 304	12.7	12.7	0

## Data Availability

Not applicable.

## Nomenclature

a	Radius of pump beam [m]
$J_{0}$	Bessel function of the first kind
k	Thermal conductivity [W/m-K]
$L_{abs}$	Optical absorption length [m]
N	Refractive index
P	Power of pump beam [W]
Q	Heat source [W/m^3^]
R	Reflectivity
T	Temperature [K]
t	Time [s]
Greek letter	
α	Thermal diffusivity [m/s]
β	Integration variable
λ	Optical absorption coefficient [1/m]
Φ	Deflection angle [°]
ψ	Phase delay [°]
ω	Angular frequency [1/s]

## Appendix A

This appendix describes the process of solving differential equations using the Hankel transformation. The complex transformation method is applied to Equation (8) using Equation (A1), it is converted to a steady-state heat conduction equation as Equations (A2)–(A5).

(A1) $$T\left(r,z,t\right)=\tilde{T}\left(r,z\right)e^{i\omega t}$$

(A2) $$\nabla^{2}{\tilde{T}}_{0}-\frac{i\omega }{\alpha_{0}}{\tilde{T}}_{0}=0$$

(A3) $$\nabla^{2}{\tilde{T}}_{1}-\frac{i\omega }{\alpha_{1}}{\tilde{T}}_{1}=-\frac{\lambda_{1}P_{1}}{4\pi k_{1}\alpha^{2}}e^{-\frac{r^{2}}{a^{2}}-\lambda_{1}z}$$

(A4) $$\nabla^{2}{\tilde{T}}_{2}-\frac{i\omega }{\alpha_{2}}{\tilde{T}}_{2}=-\frac{\lambda_{2}P_{2}}{4\pi k_{2}\alpha^{2}}e^{-\frac{r^{2}}{a^{2}}-\lambda_{2}\left(z-L_{1}\right)-\lambda_{1}L_{1}}$$

(A5) $$\nabla^{2}{\tilde{T}}_{3}-\frac{i\omega }{\alpha_{3}}{\tilde{T}}_{3}=0$$

Equations (A2)–(A5) using the complex transformation method are composed of functions of r and z. To transform this into a function of z only, it is applied to Equation (A7) to apply the Hankel transformation as in Equation (A6).

(A6) $$t_{i}\left(\beta ,z\right)=\int_{0}^{\infty }{\tilde{T}}_{i}\left(r,z\right)rJ_{0}\left(\beta r\right)dr$$

(A7) $$\nabla^{2}{\tilde{T}}_{i}=\frac{\partial^{2}{\tilde{T}}_{i}}{\partial r^{2}}+\frac{1}{r}\frac{\partial {\tilde{T}}_{i}}{\partial r}+\frac{\partial^{2}{\tilde{T}}_{i}}{\partial z^{2}}$$

The solution for each term in Equation (A7) is as Equations (A8)–(A11) below.

(A8) $$\int_{0}^{\infty }\frac{\partial^{2}{\tilde{T}}_{i}}{\partial r^{2}}rJ_{0}\left(\beta r\right)dr=-{\left[{\tilde{T}}_{i}J_{0}\left(\beta r\right)\right]}_{0}^{\infty }-\int_{0}^{\infty }{\tilde{T}}_{i}\beta J_{1}\left(\beta r\right)dr-\int_{0}^{\infty }{\tilde{T}}_{i}\beta^{2}rJ_{0}\left(\beta r\right)dr$$

(A9) $$\int_{0}^{\infty }\frac{1}{r}\frac{\partial {\tilde{T}}_{i}}{\partial r}rJ_{0}\left(\beta r\right)dr={\left[{\tilde{T}}_{i}J_{0}\left(\beta r\right)\right]}_{0}^{\infty }+\int_{0}^{\infty }{\tilde{T}}_{i}\beta J_{1}\left(\beta r\right)dr$$

(A10) $$\int_{0}^{\infty }\frac{\partial^{2}{\tilde{T}}_{i}}{\partial z^{2}}rJ_{0}\left(\beta r\right)dr=\frac{\partial^{2}t_{i}}{\partial z^{2}}$$

(A11) $$\int_{0}^{\infty }e^{-\frac{r^{2}}{a^{2}}-\lambda_{1}z}rJ_{0}\left(\beta r\right)dr=\frac{a^{2}}{2}e^{-\lambda_{1}z-\frac{a^{2}\beta^{2}}{4}}$$

By substituting Equations (A8)–(A11) into Equations (A2)–(A5) converted to a steady-state equation through a complex transformation method, the temperature distribution equation for each region is derived as shown in Equations (A12)–(A15) below.

(A12) $$\nabla^{2}{\tilde{T}}_{0}-\frac{i\omega }{\alpha_{0}}{\tilde{T}}_{0}=0\;\to \;\frac{\partial^{2}t_{0}}{\partial z^{2}}-\left(\beta^{2}+\frac{i\omega }{\alpha_{0}}\right)t_{0}=0$$

(A13) $$\nabla^{2}{\tilde{T}}_{1}-\frac{i\omega }{\alpha_{1}}{\tilde{T}}_{1}=-\frac{\lambda_{1}P_{1}}{4\pi k_{1}\alpha^{2}}e^{-\frac{r^{2}}{a^{2}}-\lambda_{1}z}\;\to \;\frac{\partial^{2}t_{1}}{\partial z^{2}}-\left(\beta^{2}+\frac{i\omega }{\alpha_{1}}\right)t_{1}=-\frac{\lambda_{1}P_{1}}{8\pi k_{1}}e^{-\frac{\beta^{2}a^{2}}{4}-\lambda_{1}z}$$

(A14) $$\nabla^{2}{\tilde{T}}_{2}-\frac{i\omega }{\alpha_{2}}{\tilde{T}}_{2}=-\frac{\lambda_{2}P_{2}}{4\pi k_{2}\alpha^{2}}e^{-\frac{r^{2}}{a^{2}}-\lambda_{2}\left(z-L_{1}\right)-\lambda_{1}L_{1}}\;\to \;\frac{\partial^{2}t_{2}}{\partial z^{2}}-\left(\beta^{2}+\frac{i\omega }{\alpha_{2}}\right)t_{2}=-\frac{\lambda_{2}P_{2}}{8\pi k_{2}}e^{-\frac{\beta^{2}a^{2}}{4}-\lambda_{2}\left(z-L_{1}\right)-\lambda_{1}L_{1}}$$

(A15) $$\nabla^{2}{\tilde{T}}_{3}-\frac{i\omega }{\alpha_{3}}{\tilde{T}}_{3}=0\;\to \;\frac{\partial^{2}t_{3}}{\partial z^{2}}-\left(\beta^{2}+\frac{i\omega }{\alpha_{3}}\right)t_{3}=0$$

Equations (A16)–(A19) to solve the homogeneous solution and the particular solution of the temperature distribution equation for each region in Equations (A12)–(A15) are same as Equations (A16)–(A19) below.

(A16) $$\begin{array}{l} \frac{\partial^{2}t_{0}}{\partial z^{2}}-\left(\beta^{2}+\frac{i\omega }{\alpha_{0}}\right)t_{0}=0\;\leftarrow \;\mathrm{if}\;t_{0}=Ae^{fz},\;\frac{\partial^{2}t_{0}}{\partial z^{2}}=f^{2}t_{0}\\ \to \;t_{0}=C_{1}e^{\delta_{0}z}+C_{2}e^{-\delta_{0}z} \end{array}$$

(A17) $$\begin{array}{c} \frac{\partial^{2}t_{1}}{\partial z^{2}}-\left(\beta^{2}+\frac{i\omega }{\alpha_{1}}\right)t_{1}=-\frac{\lambda_{1}P_{1}}{8\pi k_{1}}e^{-\frac{\beta^{2}a^{2}}{4}-\lambda_{1}z}\;\leftarrow \;\delta_{1}=\sqrt{\beta^{2}+\frac{i\omega }{\alpha_{1}}}\\ -\;\mathrm{Homogeneous}\;\mathrm{solution}\\ {\left(t_{1}\right)}_{h}=C_{3}e^{\delta_{1}z}+C_{4}e^{-\delta_{1}z}\\ -\;\mathrm{Particular}\;\mathrm{solution}\\ \mathrm{Assumed}\;\mathrm{of}\;\mathrm{right}\;\mathrm{side}\;ke^{ex}\;\mathrm{form},\;{\left(t_{1}\right)}_{p}=C_{p}e^{-\lambda_{1}z},\\ \frac{\partial^{2}{\left(t_{1}\right)}_{p}}{\partial z^{2}}=C_{p}\lambda_{1}^{2}e^{-\lambda_{1}z}=\lambda_{1}^{2}{\left(t_{1}\right)}_{p}\\ \to t_{1}=C_{3}e^{\delta_{1}z}+C_{4}e^{-\delta_{1}z}+A_{1}e^{-\lambda_{1}z}\;at\;A_{1}=\frac{\lambda_{1}P_{1}e^{-\frac{\beta^{2}a^{2}}{4}}}{8\pi k_{1}\left(\lambda_{1}^{2}-\delta_{1}^{2}\right)} \end{array}$$

(A18) $$\begin{array}{c} \frac{\partial^{2}t_{2}}{\partial z^{2}}-\left(\beta^{2}+\frac{i\omega }{\alpha_{2}}\right)t_{2}=-\frac{\lambda_{2}P_{2}}{8\pi k_{2}}e^{-\frac{\beta^{2}a^{2}}{4}-\lambda_{2}\left(z-L_{1}\right)-\lambda_{1}L_{1}}\;\leftarrow \;\delta_{2}=\sqrt{\beta^{2}+\frac{i\omega }{\alpha_{2}}}\\ -\;\mathrm{Homogeneous}\;\mathrm{solution}\\ {\left(t_{2}\right)}_{h}=C_{5}e^{\delta_{2}\left(z-L_{1}\right)}+C_{6}e^{-\delta_{2}\left(z-L_{1}\right)}\\ -\;\mathrm{Particular}\;\mathrm{solution}\\ \mathrm{Assumed}\;\mathrm{of}\;\mathrm{right}\;\mathrm{side}\;ke^{ex}\;\mathrm{form},\;{\left(t_{2}\right)}_{p}=C_{p}e^{-\lambda_{2}\left(z-L_{1}\right)},\\ \frac{\partial^{2}{\left(t_{2}\right)}_{p}}{\partial z^{2}}=C_{p}\lambda_{2}^{2}e^{-\lambda_{2}\left(z-L_{1}\right)}=\lambda_{2}^{2}{\left(t_{2}\right)}_{p}\\ \to t_{2}=C_{5}e^{\delta_{2}\left(z-L_{1}\right)}+C_{6}e^{-\delta_{2}\left(z-L_{1}\right)}+A_{2}e^{-\lambda_{2}\left(z-L_{1}\right)}\;at\;A_{2}=\frac{\lambda_{2}P_{2}e^{-\frac{\beta^{2}a^{2}}{4}-\lambda_{1}L_{1}}}{8\pi k_{2}\left(\lambda_{2}^{2}-\delta_{2}^{2}\right)} \end{array}$$

(A19) $$\begin{array}{l} \frac{\partial^{2}t_{3}}{\partial z^{2}}-\left(\beta^{2}+\frac{i\omega }{\alpha_{3}}\right)t_{3}=0\;\leftarrow \;\mathrm{if}\;t_{3}=Ae^{fz},\;\frac{\partial^{2}t_{3}}{\partial z^{2}}=f^{2}t_{3}\\ \to \;t_{3}=C_{7}e^{\delta_{3}\left(z-L_{1}-L_{2}\right)}+C_{8}e^{-\delta_{3}\left(z-L_{1}-L_{2}\right)} \end{array}$$

In the case of regions 0 and 3 (Air region), there is no change in temperature as the distance from the specimen increases, Equations (A16) and (A19) can be expressed as below.

$$\underset{z\to -\infty }{\lim}\frac{\partial t_{0}}{\partial z}=0\;\to \;\underset{z\to -\infty }{\lim}\left(C_{1}e^{\delta_{0}z}+C_{2}e^{-\delta_{0}z}\right)=0,\;C_{2}=0\;\to \;∴t_{0}=C_{1}e^{-\delta_{0}z}$$

$$\underset{z\to \infty }{\lim}\frac{\partial t_{3}}{\partial z}=0\;\to \;\underset{z\to \infty }{\lim}\left(C_{7}e^{\delta_{3}\left(z-L_{1}-L_{2}\right)}+C_{8}e^{-\delta_{3}\left(z-L_{1}-L_{2}\right)}\right)=0,\;C_{7}=0\;\to \;∴t_{3}=C_{8}e^{-\delta_{3}\left(z-L_{1}-L_{2}\right)}$$

In the case of regions 1 and 2 (specimen region), Equations (A17) and (A18) can be expressed as below using $\cosh\theta =\frac{e^{\theta }+e^{-\theta }}{2}$, $\sinh\theta =\frac{e^{\theta }-e^{-\theta }}{2}$.

$$t_{1}=A_{1}e^{-\lambda_{1}z}+C_{3}\cosh\left(\delta_{1}z\right)+C_{3}\sinh\left(\delta_{1}z\right)$$

$$t_{2}=A_{2}e^{-\lambda_{2}\left(z-L_{1}\right)}+C_{5}\cosh\left(\delta_{2}\left(z-L_{1}\right)\right)+C_{6}\sinh\left(\delta_{2}\left(z-L_{1}\right)\right)$$

The boundary condition is converted as shown in Equation (A20) below.

(A20) $$\begin{array}{cc} t_{0}=t_{1}\;at\;z=0 & k_{0}\frac{\partial t_{0}}{\partial z}=k_{1}\frac{\partial t_{1}}{\partial z}\;at\;z=0\\ t_{1}=t_{2}\;at\;z=L_{1} & k_{1}\frac{\partial t_{1}}{\partial z}=k_{2}\frac{\partial t_{2}}{\partial z}\;at\;z=L_{1}\\ t_{2}=t_{3}\;at\;z=L_{1}+L_{2} & k_{2}\frac{\partial t_{2}}{\partial z}=k_{3}\frac{\partial t_{3}}{\partial z}\;at\;z=L_{1}+L_{2} \end{array}$$

By substituting the boundary conditions for the unknowns of the temperature distribution equation in each region, the Hankel transformation is finally completed. Equations (15)–(18) can be obtained by performing the reverse Hankel transformation using the governing Equations (A16)–(A19) and the boundary condition Equation (A20).

## Appendix B

### Appendix B1. Uncertainty Analysis

In this study, uncertainty analysis was conducted in accordance with ISO/IEC Guide 98-3:2008, Uncertainty of measurement—part 3 Guide to the expression of uncertainty in measurement (GUM: 1995. SUPPL 2: 2011) as mentioned by Coleman et al. [45,46].

According to the ISO Guide, the combined standard uncertainty is calculated using the sensitivity coefficient or the relative standard uncertainty after performing type A evaluation of standard uncertainty and type B evaluation of standard uncertainty. According to the ISO Guide, the combined standard uncertainty is calculated using the sensitivity coefficient or the relative standard uncertainty after performing type A evaluation of standard uncertainty and type B evaluation of standard uncertainty. Then, coverage factor is calculated through the effective degree of freedom, which is calculated by using the uncertainty estimation model from the combined standard uncertainty and then figuring out of expended uncertainty.

Type A evaluation of standard uncertainty is a method of estimating the repeatability of a measurement by using the value of the positive square root of the experimental variance as the standard deviation of the mean value by repeated measurements as a component resulting from the random error occurring during the measurement process. Type B evaluation of standard uncertainty is an evaluation method based on information representative of systematic errors such as past measurement data, experience or general knowledge about the behavior or characteristics of materials and equipment used in experiments, and data given in calibration and other certificates.

### Appendix B2. Uncertainty Analysis for Phase Delay of Pump and Probe Beam

In this study, the thermal conductivity of three materials were determined by measuring the phase delay of the pump and probe beam. Therefore, uncertainty analysis was performed on the phase delay measurement. The following Table A1 lists the input quantity set for the uncertainty analysis of the phase delay.

**Table A1:** Input quantity for uncertainty analysis.

Category	Input Quantity
Frequency of pump beam (Hz)	20
Diameter of pump beam (μm)	70
Relative position (mm)	0
Height of probe beam (μm)	150
Environment temperature (°C)	25
Thermal conductivity of air (W/m-K)	0.02624

The type A evaluation of standard uncertainty for the phase delay of the pump and probe beam is the standard deviation of the measured values in repeated measurements caused by random errors. Among the aforementioned input values, it was estimated that the ambient temperature and the thermal conductivity according to the temperature were included in the random error in the phase delay measurement. The measurement of phase delay is defined as the average of phase delays measured for 30 s at 0.1 s intervals during one point measurement. Therefore, it can be judged as a well-characterized measurement under statistical control, and in this case, the joint estimate $\left(s_{p}^{2}\right)$ can be used for the type A evaluation of standard uncertainty as in Equation (A21).

(A21) $$s_{p}^{2}=\frac{\sum_{i=1}^{N}v_{i}s_{i}^{2}}{\sum_{i=1}^{N}v_{i}}$$

Here, $s_{i}^{2}$ is the experimental variance of the i-th $n_{i}$ independent observations, and the degree of freedom is $v_{i}=n-1$. Using a joint estimate, the type A evaluation of standard uncertainty is $u=s_{p}/\sqrt{n}$.

The type B evaluation of standard uncertainty of the deflection angle to the probe beam is the estimated variance ($u^{2}\left(x_{i}\right)$) or the standard uncertainty ($u\left(x_{i}\right)$) for the estimated value ($x_{i}$) of the input quantity ($X_{i}$), not obtained through repeated measurements.

Systematic factors of uncertainty about the phase delay of the pump and probe beam are caused by the motorized stage that adjusts the relative position, the mechanical chopper, the PSD sensor, the beam profiler, and the Lock-in amplifier. The uncertainty of systematic factor was calculated through the specifications of the experimental equipment mentioned in 3.2 Experimental setup, and the probability distribution was assumed to be a rectangular distribution. Assuming that the correlation between the uncertainty and the phase delay due to the probability distribution is in the form of a linear sum, the combined standard uncertainty ($u_{c}\left(y\right)$) can be expressed as the sum of squares of the type A evaluation of standard uncertainty and the type B evaluation of standard uncertainty as shown in Equation (A22) below.

(A22) $$u_{c}\left(y\right)=\sqrt{\overset{N}{\underset{i=1}{\sum }}u^{2}\left(x_{i}\right)}$$

The expanded uncertainty (U) is obtained by multiplying the combined standard uncertainty by the coverage factor ($k_{c}$) as shown in Equation (A23) below.

(A23) $$U=k_{c}u_{c}\left(y\right)$$

Here, the coverage factor is usually taken as $k_{c}=2$ from a confidence level of about 95%.

Table A2 below shows the uncertainty analysis results derived based on 360°, the maximum value that can measure the phase delay in this study.

**Table A2:** Results of Uncertainty analysis.

Category		Value
Type A evaluation of standard uncertainty (°)		0.0591
Type B evaluation of standard uncertainty (°)	Mechanical chopper	4.1569
	PSD sensor	2.0785
	Lock-in Amplifier	2.0785
	Motorized stage	0.2078
	Beam profiler	4.1569
Combined standard uncertainty (°)		6.5762
Expanded uncertainty (°)		13.2
Uncertainty of phase delay (%)		3.7

Finally, in this study, the measured phase delay is 360° ± 13.2° at the maximum phase delay value 360° for uncertainty analysis. The expanded uncertainty was reported that the composite standard uncertainty was multiplied by the coverage factor $k_{c}=2$ obtained from a confidence level of about 95%. The uncertainty of the phase delay of the pump and probe beam analyzed in this study was derived as 3.7% with a confidence level about 95%.
